# Cardiac Myosarcoma in a Newborn Infant—A Case Report and Literature Review

**DOI:** 10.3389/fcvm.2021.675202

**Published:** 2021-07-14

**Authors:** Liliya Vakrilova, Rumen Marinov, Stanislava Hitrova-Nikolova, Dobri Dobrev, Maxim Denev, Stoyan Lazarov

**Affiliations:** ^1^Faculty of Medicine, Medical University of Sofia, Sofia, Bulgaria; ^2^Department of Neonatology, University Obstetrics and Gynecology Hospital “Maichin Dom”, Sofia, Bulgaria; ^3^Department of Pediatric Cardiology, National Cardiac Hospital, Sofia, Bulgaria

**Keywords:** cardiomyosarcoma, congenital malignant tumors, echocardiography, newborn infants, cardiac surgery, congenital heart disease

## Abstract

**Background:** Malignant cardiac neoplasms are extremely rare in neonates. Prenatal diagnosis is often not available. Initial symptoms can mimic non-cardiac diseases. We present a pre-mature newborn, birth-weight 2,480 g, 34 gestational weeks, which underwent cardiac surgery due to a cardiac tumor.

**Case Summary:** This was a 3–rd pregnancy after two spontaneous abortions to a mother with thrombophilia, diabetes, hydramnios, and retroplacental hematoma. The baby was admitted to NICU with transitory respiratory failure and inborn infection; hence oxygen-supplementation and antibiotics were initiated. On day 11 a deterioration with tachypnea, high oxygen requirements, significantly increasing C-reactive protein values were noted. Chest radiographs were unremarkable. On day 18 a life-threatening condition with clinical symptoms of shock was identified. Echocardiography showed a large tumor formation in the right atrium, reduced blood flow in the right ventricle and pulmonary artery. On day 19 cardiac surgery was performed: a large tumor with a myxomatous appearance that occupied the cavity of the right atrium and infiltrated the annulus of the tricuspid valve was extirpated. The front wall was restored with a patch. Hemodynamics was temporarily stabilized. On the following day, ventricular fibrillation and asystole occurred. Despite life support efforts, the outcome was lethal. The histological result confirmed leiomyosarcoma of the right atrium and right ventricular hypotrophy.

**Conclusion:** Inborn cardiac sarcomas are extremely rare. The prognosis is poor. Due to fast progression in the third trimester, they can be missed by fetal echocardiography in earlier pregnancy. The postnatal clinical course is aggressive, not specific until invasive infiltration or obstruction by the tumor mass occurred. In our case, the sarcoma mimicked an inborn infection, followed by nosocomial infection and septic shock. Despite low incidence, cardiac tumors should be kept in mind and echocardiography should be conducted if there are unclear symptoms with progressive deterioration during the neonatal period.

## Introduction

Primary cardiac tumors are rare in the fetus and newborn infant. In a large series of 14,000 fetal echocardiographic examinations over 8 years an incidence of 0.14% was established. Most of them were benign, mainly rhabdomyomas, 89% (1). Primary malignant cardiac neoplasms are considerably rarer in neonates. Prenatal diagnosis is often not available, especially if fetal echocardiography was not performed during pregnancy. Initial symptoms can mimic other cardiac and non-cardiac diseases. That makes prenatal or early postnatal diagnosis difficult. Only a few cases of inborn malignant tumors have been reported, with the predominance of rhabdomyosarcoma (1–4). Here we report a late pre-term newborn who underwent cardiac surgery due to a malignant cardiac tumor.

## Case Presentation

This was a 3–rd pregnancy after two spontaneous abortions to a mother with thrombophilia and gestational diabetes. Routine follow-up of the pregnancy did not reveal any pathological findings in the fetal development. The ultrasound examination of the fetus at 20 weeks of gestation found no abnormalities of the fetal morphology, but fetal echocardiography was not performed. Hydramnios, and retroplacental hematoma were diagnosed shortly before delivery. A cesarean section was performed due to fetal distress at 34(+5) weeks of gestation: the baby was female with birth weight 2,480 g and Apgar scores at 1st min = 6 and 5th min = 7; umbilical artery pH =7.23, base excess = −14. She was admitted to the Neonatal intensive care unit (NICU) after delivery because of transitory respiratory failure, requiring brief oxygen supplementation. Laboratory data showed signs of inborn infection: slightly elevated C-reactive protein (CRP) values, thus antibiotic therapy was initiated. During the first days of life, the condition of the baby was stable, but CRP values rose rapidly - 33.7 mg/L on the day (D) 5, and 146 mg/L on D 9, and remained high thereafter. On D11 a progressive clinical deterioration was noted: tachypnea, increase in the oxygen requirements, decreased reactivity. *Serratia marcescens* and *Klebsiella pneumoniae* (D 9), *Enterococcus* spp., and Staphylococcus epidermidis (D15) were isolated from peripheral specimens. Broad-spectrum antibiotics were initiated according to the established sensitivity. Chest radiographs were unremarkable.

**Figure 1:**
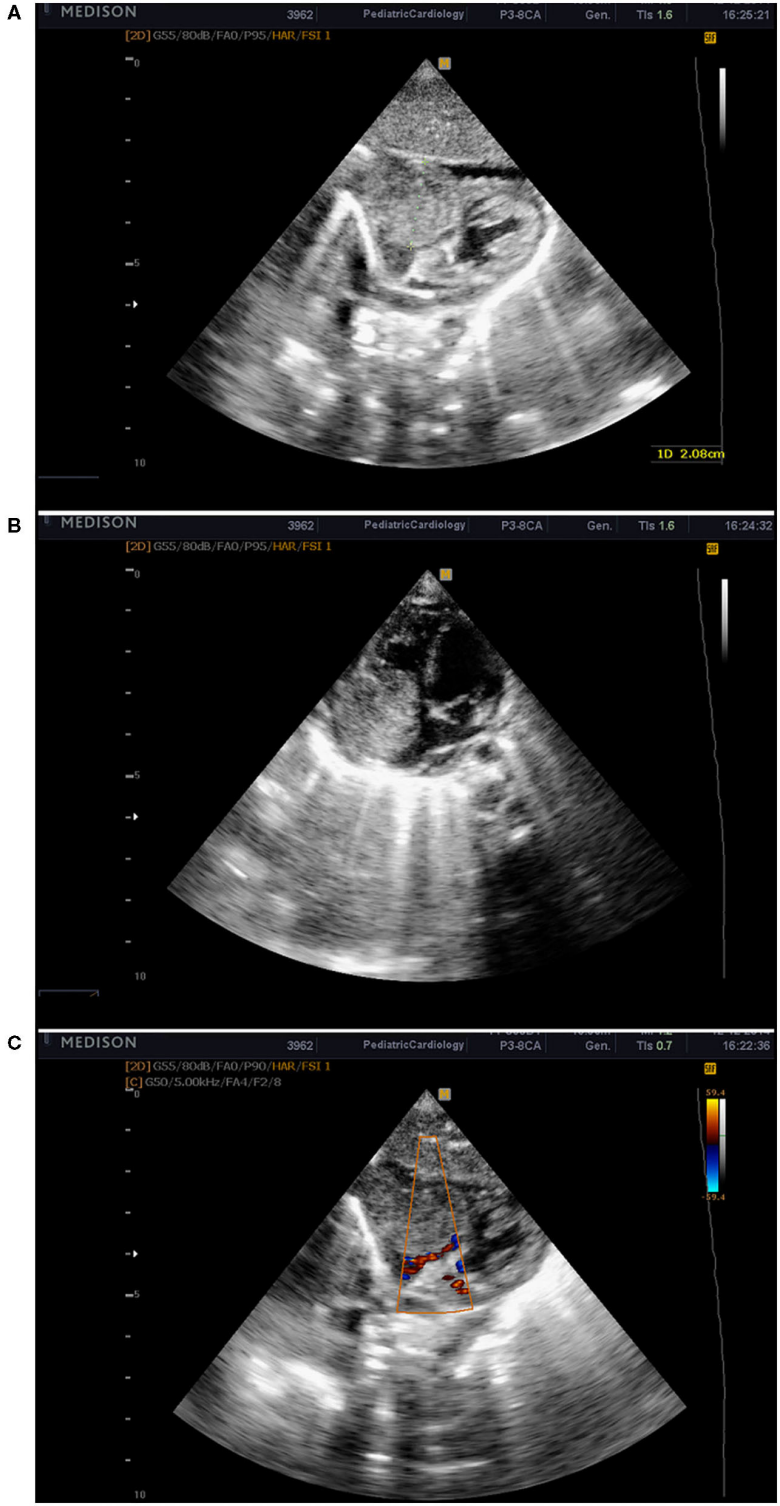
Echocardiographic images from the subcostal window showing the tumor mass in the right atrium **(A,B)**. Obstruction in the output of the right atrium and reduced blood flow to the right ventricle **(C)**.

On D18 a life-threatening deterioration with clinical symptoms of shock occurred, the baby was intubated, and mechanical ventilation was initiated. Echocardiography was performed and showed a large tumor formation in the right atrium 2 × 2.3 cm, reduced blood flow into the right ventricle and pulmonary artery, and minimal pericardial effusion (Figure 1). The patient was immediately transferred to the pediatric cardiology clinic and admitted with unstable hemodynamic parameters, on conventional mechanical ventilation (Synchronized Intermittent Positive Pressure Ventilation, FiO_2_ 1.0) with arterial oxygen saturation of 74%, along with moderately increased lactate levels; an enlarged liver was palpated up to the level of the umbilical horizontal line. These data suggested impaired systemic venous drainage. An additional echocardiographic examination was performed and revealed an infiltration growth of the tumor to the tricuspid valve and the right ventricle. The enormous tumor formation filled the entire right atrial cavity. Our first hypothesis was that the tumor formation was a myxoma of the right atrium, with a differential diagnosis for rhabdomyoma or thrombus. The case was analyzed and discussed with pediatric cardiologists and cardiac surgeons. Based on the data known so far and the hemodynamic instability, a decision was made for immediate surgery. Pre-operative echocardiography also demonstrated an atrial septum deviation toward the left atrium and a Foramen ovale communication with a right-to-left shunting. Pulsed Wave Doppler (PW Doppler) velocity through the tricuspid valve was 1.4 m/s with a mean RA-RV gradient of 4 mm. PW Doppler velocity across the pulmonary valve was 0.6 m/s.

On D 19 cardiac surgery was performed. The onset of surgery was in the setting of hemodynamically unstable condition and impaired venous drainage. Upon incision of the pericardium and proximally to the atrio-ventricular (AV) groove a large tumor formation with a myxomatous appearance, “the size of an olive,” was found. The formation infiltrated the myocardium wall of the right ventricle. After right atriotomy the tumor formation was described as occupying the entire cavity of the right atrium, excluding the right atrial auriculum, and obstructing the tricuspid valve. The formation infiltrated the tricuspid valve annulus too. A total extirpation of the tumor mass was performed. Additionally, a portion of the associated right atrial anterior wall and, as much as possible, the tumor infiltration in the tricuspid annulus and right ventricular myocardium were resected. During the surgery, the posterior cusp of the tricuspid valve was detached and subsequently restored into the annulus. The atrial communication (Foramen ovale) was left open. The front wall of the right atrium was restored with a patch. The operation was finalized with an open sternum. The post-operative echo showed no obstruction at the level of the tricuspid valve, moderate tricuspid insufficiency and left-to-right shunt through the atrial communication (foramen ovale). During the early post-operative period the patient required maximum inotropic support under conventional mechanical ventilation to maintain stable hemodynamic parameters. At the 12th h after the operation (D20) a sudden critical deterioration with ventricular fibrillation (VF) and asystole occurred. Despite the initiated cardiopulmonary resuscitation and life support measures, the outcome was lethal. Table 1 summarizes the clinical presentation and management of the patient.

**Table 1:** Timeline of events.

**Days (D)**	**Event**
Birth	• Cesarean section • Birthweight 2,450 g • 34^(+5)^ weeks of gestation
D5-D9	• Progressive increase in CRP levels • Nosocomial infection suspected • Antibiotics added
From D11	• Progressive respiratory falure
D18	• Critical deterioration • Echocardiogram: - large formation in the right atrium - reduced blood flow into the right ventricle and pulmonary artery • Transfer to the pediatric cardiology ward
D19	• Cardiac surgery: - Tumor formation that occupied the cavity of the right atrium and infiltrated the ring of the tricuspid valve was extirpated - Hemodynamics: temporarily stabilized
D20	• Ventricular fibrillation and asystole • Exitus letalis despite resuscitation eforts

**Figure 2:**
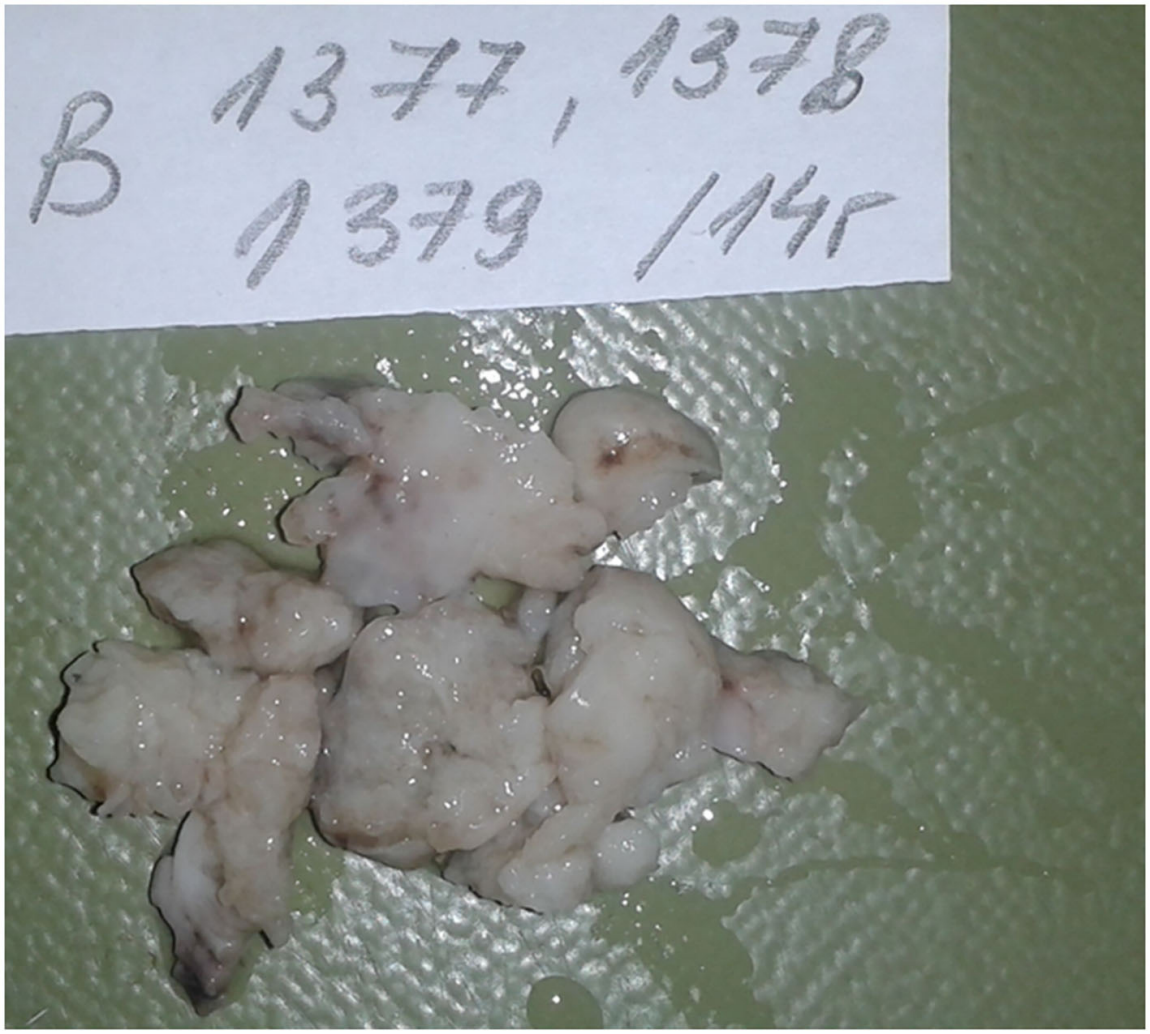
Macroscopic appearance of the extirpated tumor formation.

**Figure 3:**
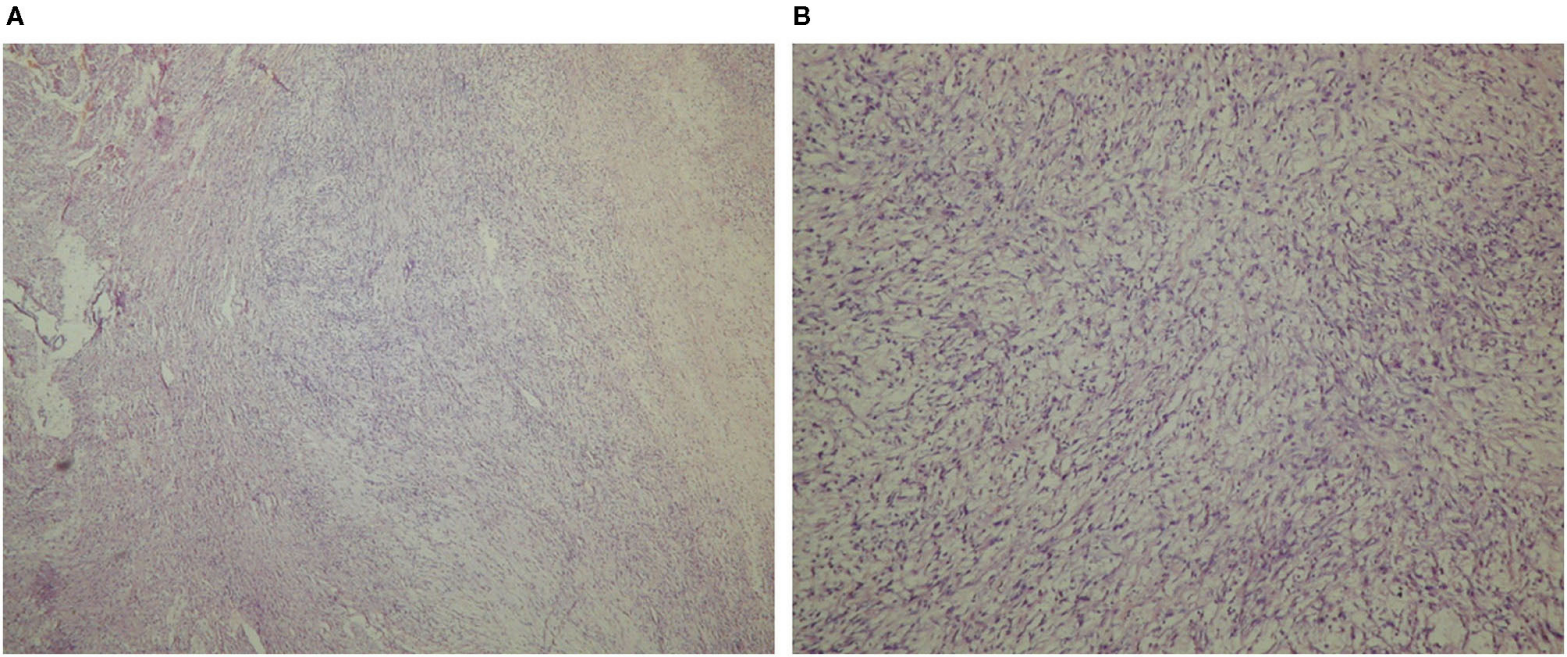
Histologic specimen acquired from the tumor mass, light microscopic examination (Hematoxylin-Eosin stain), 40× magnification **(A)**, and 100× magnification **(B)**.

The pathological assessment of the intraoperatively extirpated tumor described a gross structure of lobulated masses with white-pink translucent tissue, with a myxomatous appearance and soft flexible texture (Figure 2). The histomorphology of the tumor mass showed a malignant neoplasm with the structure of a moderately differentiated sarcoma and zones of necrosis and hemorrhage. Tumor periphery had signs of infiltration into atrial wall. These findings corresponded to leiomyosarcoma (Figure 3). A differential diagnosis was made with a fibrosarcoma. On post-mortem microscopic histomorphology inspection of specimens from the atria and ventricles hemorrhage and ischemic zones were found in the myocardium and conduction system (the bundle and distal branches). No specimen showed tumor infiltration in these structures.

## Discussion

Primary cardiac tumors are very rare. They are found in ~1 in 10 000 (0.01%) of routine post-mortem examinations of patients of all ages with an incidence ranging between 0.0017 and 0.3% according to data from autopsy series published in various studies (5–7). Yu et al. reviewed 33,108 cases of cardiac operations in all age groups performed for a period of 10 years (1996–2005). Among them, 234 cases were confirmed histologically as primary cardiac tumors, which represents 0.71% of the group. The majority of them were benign - 90.6% (8). About 25% of all primary cardiac tumors occur in childhood (6, 9). A retrospective review conducted in the late 1990s detected cardiac tumors in < 0.5% of 30,000 children under evaluation for cardiac diseases (10). The majority of the cases and case series published in the literature report a high frequency of benign primary cardiac neoplasms 90–97%, and <10% malignancy (7, 11–13).

The incidence of cardiac tumors diagnosed by fetal echocardiography has been reported to be ~0.14% (1). Beghetti et al. studied a pediatric population of 27,640 patients assessed for cardiac diseases for 15 years, and diagnosed 56 patients with primary cardiac tumors, in 12 of them the diagnosis was established before birth. Due to improved diagnostic and imaging capabilities, an increasing incidence of primary cardiac tumors was found: 0.06% for the period 1980–84; 0.22% for 1985–89, and 0.32% for 1990–95 (10). Despite many years of experience, most knowledge of this disease is based on case reports and case series rather than large cohort studies.

The types of heart tumors in the pediatric population differ from those seen in adults. In the latter, cardiac myxomas are the most common tumors (8, 14). In infants, the most common primary tumor of the heart is the rhabdomyoma (44–78%), followed by fibroma, teratoma, vascular tumors, and myxoma (10, 15–17). So, any intracavitary mass in infants is suggestive of a cardiac rhabdomyoma unless proven otherwise (14, 18). There is a high incidence of associated tuberous sclerosis among infants with rhabdomyoma, ~50–86%, that's why all neonates and fetuses with suspected rhabdomyoma need further genetic examination for Tuberous Sclerosis (13, 17, 19, 20).

Malignant variants of primary heart tumors in fetus and neonate occur, but they are exceedingly rare, only single cases are published in the literature. In this study, we report a newborn infant with cardiac sarcoma which occupied the cavity of the right atrium and infiltrated the tricuspid valve. An antenatal diagnosis was not available. After birth, there were no clinical symptoms of cardiac disease, so the diagnosis was not suspected until a critical deterioration with symptoms of cardiac arrest due to obstruction to blood flow caused by the rapid and invasive tumor growth occurred.

Isaacs et al. analyzed the clinical findings, imaging studies, pathology, and outcome of 224 fetuses and neonates with primary cardiac tumors collected from the literature: 53.6% of them were rhabdomyomas, and only 2% were malignant (16). In the study of Linnemayer et al. over 45 years, 64 patients <18 years were diagnosed with cardiac tumors and were evaluated for surgical resection. Seventeen percentage (*n* = 11) of the tumors showed malignant characteristics (13). Shi et al. analyzed the medical records of pediatric patients with cardiac tumors at four hospitals. Among the 158 cases of primary cardiac tumors, 150 were benign and eight (5%) were malignant (11). According to the results of these different studies, about 2–17% of the cardiac tumors in infancy show malignancy with angiosarcoma and rhabdomyosarcoma being the most common, followed by fibrosarcoma and lymphoma (9, 11, 13, 16, 21). Cardiosarcomas are mesenchymal, usually single tumors of variable morphology, and represent 95% of the primary cardiac malignancies in all age groups. They often give metastases in the lungs and mediastinum, which worsens the prognosis. Rhabdomyosarcomas are the second most common primary malignancies of the heart; their origin is of striated muscle. They rarely invade beyond the pericardium. The postnatal clinical course of the cardiac sarcomas is aggressive, often not specific until severe local infiltration and intracavitary obstruction is present. In most patients the outcome is lethal (2). The clinical manifestations are variable and include recurrent pericardial effusions, respiratory distress, arrhythmia, heart failure.

On magnetic resonance imaging, sarcomas present as heterogeneous invasive masses with hemorrhage, necrosis, valve destruction, extra-cardiac growth, and metastases (22, 23). Cardiac malignant tumors are best diagnosed by bi-dimensional echocardiography (10, 23). Fetal echocardiography can help in the early diagnosis. Due to fast progression in the third trimester, malignant cardiac tumors can be missed during fetal echocardiography at earlier stages of pregnancy. Isolated cases can be diagnosed as early as 20 weeks of gestation but the majority of the sarcomas develop later in pregnancy and become detectable in the third trimester if fetal echocardiography is performed periodically (9).

We consider that fetal echography in the last months of pregnancy, while absent in our case, would contribute to timely discussion of the case, performing additional diagnostic tests, and decision-making for pre- and postnatal treatment. Our newborn infant had the clinical and laboratory findings of inborn, followed by nosocomial infection, so we decided that was the reason for the unstable condition in the NICU. The main symptoms were those associated with mild to moderate respiratory failure. Only after the critical deterioration with symptoms of shock occurred, we focused on differential diagnosis with cardiac disease. Therefore, the echocardiography was performed at D18. Hemodynamic instability with low cardiac output, impaired systemic venous drainage and the presence of massive tumor formation were the indications for urgent surgery in our case. Undoubtedly, additional imaging studies would have been extremely useful for clarifying in details the characteristics of the formation and planning of timing and extensiveness of the surgery. The cardiac MRI before surgery would greatly improve the diagnostics, and clarify the course and prognosis in such pathology. The causes of the abrupt deterioration and VT that determined the outcome for our patient remained debatable. Since pathomorphological samples of atrial and ventricular muscle (post-mortem) showed no residual tumor infiltration, we can conclude that the malignant neoplasm was resected almost completely. On the other hand, hemorrhage and ischemic zones were found in the myocardium and conduction system, so we can suggest that the surgery and the extensive tumor resection rather than the tumor itself might have triggered the fatal ventricle dysrhythmia. Because of the lack of specific symptoms during the neonatal period, if there is no antenatal diagnosis, as was the case described here, in most of them the diagnosis is made when the disease progresses and severe complications occur. The prognosis of such inborn malignant tumors is poor with a low survival rate and frequent recurrences, and also depends on the histological subtype and the degree of atypicality. Surgical resection of the tumor, chemotherapy, and radiotherapy may prolong survival (1, 9).

Only single cases with early prenatal or postnatal diagnosis followed by successful surgical intervention and treatment are reported. Ludomirsky et al. (24), describes a case of a 3-month-old female infant with the physical findings of grade 3/6 harsh systolic ejection murmur at the upper left sternal border noted on routine examination. Echocardiography revealed a tumor mass in the right ventricle, which was completely surgically removed, with a histologic diagnosis of undifferentiated sarcoma. The post-operative course was unremarkable, but there was no data for long-term follow-up (24). Mukai et al. reported a case of Congenital infantile fibrosarcoma detected by prenatal ultrasonography at 24 weeks' gestation. The baby was delivered by Cesarean section at 27 weeks' gestation. Successful complete tumor resection was performed on day 3. At the age of 2.5 years, the infant was healthy without evidence of recurrence. Because cases with congenital infantile sarcoma usually have a poor prognosis, termination of pregnancy at an appropriate time, or postnatal early treatment to improve outcome are discussed (25).

## Conclusion

Despite their low incidence inborn cardiac tumors should be kept in mind in the neonatal period and infancy. Fetal follow-up and serial echocardiographic studies would allow early fetal diagnosis, appropriate decision making, and treatment. In our case, the sarcoma in the right atrium mimics an inborn infection, followed by nosocomial infection and septic shock. The diagnosis was made just after the critical deterioration occurred when the tumor had already occupied the cavity of the right atrium and penetrated to the ventricle. If there are unclear symptoms with progressive deterioration in the neonatal period echocardiography should be made to rule out or confirm cardiac disease, to ensure diagnosis and discuss treatment options before critical complications occur.

## Data Availability Statement

The raw data supporting the conclusions of this article will be made available by the authors, without undue reservation.

## Ethics Statement

Written informed consent for the publication of any potentially identifiable images or data included in this article was obtained from the patient's legal guardian/next of kin.

## Author Contributions

LV contributed to the conception and design of this case report, manuscript drafting, and a literature search. RM interpreted the data and drafted a part of the manuscript. SH-N discussed the data and edited the manuscript. DD, MD, and SL discussed the case and revised the manuscript. All authors agree to be accountable for the content of the work.

## Conflict of Interest

The authors declare that the research was conducted in the absence of any commercial or financial relationships that could be construed as a potential conflict of interest.

## References

[1] Holley DG Martin GR Brenner JI Fyfe DA Huhta JC Kleinman CS. Diagnosis and management of fetal cardiac tumors: a multicenter experience and review of published reports. J Am Coll Cardiol. (1995) 26:516–20. 10.1016/0735-1097(95)80031-B 7608458

[2] Tutak E Satar M Ozbarlas N Uguz A Yapicioglu H Narli N. A newborn infant with intrapericardial rhabdomyosarcoma: a case report. Turk J Pediatr. (2008) 50:179–81. 18664085

[3] Chan HS Sonley MJ Moës CA Daneman A Smith CR Martin DJ. Primary and secondary tumors of childhood involving the heart, pericardium, and great vessels. A report of 75 cases and review of the literature. Cancer. (1985) 56:825–36. 10.1002/1097-0142(19850815)56:<825::AID-CNCR2820560421>3.0.CO;2-7 4016674

[4] Sallee D Spector ML van Heeckeren DW Patel CR. Primary pediatric cardiac tumors: a 17 year experience. Cardiol Young. (1999) 9:155–62. 10.1017/S1047951100008374 10323513

[5] Patel J Sheppard MN. Pathological study of primary cardiac and pericardial tumours in a specialist UK Centre: surgical and autopsy series. Cardiovasc Pathol. (2010) 19:343–52. 10.1016/j.carpath.2009.07.005 19747857

[6] McAllister HA Jr. Primary tumors of the heart and pericardium. Pathol Annu. (1979) 14 Pt 2:325–55. 232754

[7] Reynen K. Frequency of primary tumors of the heart. Am J Cardiol. (1996) 77:107–16. 10.1016/S0002-9149(97)89149-7 8540447

[8] Yu K Liu Y Wang H Hu S Long C. Epidemiological and pathological characteristics of cardiac tumors: a clinical study of 242 cases. Interact Cardiovasc Thorac Surg. (2007) 6:636–9. 10.1510/icvts.2007.156554 17670730

[9] Uzun O Wilson DG Vujanic GM Parsons JM De Giovanni JV. Cardiac tumours in children. Orphanet J Rare Dis. (2007) 2:11. 10.1186/1750-1172-2-11 17331235 PMC3225855

[10] Beghetti M Gow RM Haney I Mawson J Williams WG Freedom RM. Pediatric primary benign cardiac tumors: a 15-year review. Am Heart J. (1997) 134:1107–14. 10.1016/S0002-8703(97)70032-2 9424072

[11] Shi L Wu L Fang H Han B Yang J Ma X. Identification and clinical course of 166 pediatric cardiac tumors. Eur J Pediatr. (2017) 176:253–60. 10.1007/s00431-016-2833-4 28074279

[12] Koç M Kutsal A. Rare operations in pediatric heart surgery: cardiac tumors in childhood. Turk Gogus Kalp Damar Cerrahisi Derg. (2018) 26:544–9. 10.5606/tgkdc.dergisi.2018.16147 32082795 PMC7018197

[13] Linnemeier L Benneyworth BD Turrentine M Rodefeld M Brown J. Pediatric cardiac tumors: a 45-year, single-institution review. World J Pediatr Congenit Heart Surg. (2015) 6:215–9. 10.1177/2150135114563938 25870340

[14] Becker AE. Primary heart tumors in the pediatric age group: a review of salient pathologic features relevant for clinicians. Pediatr Cardiol. (2000) 21:317–23. 10.1007/s002460010071 10865004

[15] Paramés F Freitas I Martins JD Trigo C Pinto MF. Cardiac tumors: the 17-year experience of pediatric cardiology department. Rev Port Cardiol. (2009) 28:929–40. 19998805

[16] Isaacs H Jr. Fetal and neonatal cardiac tumors. Pediatr Cardiol. (2004) 25:252–73. 10.1007/s00246-003-0590-4 15360117

[17] Dominguez C Perkins A Duque A Bravo V. Primary cardiac tumors in infancy: a case report and literature review. Acad Forensic Pathol. (2017) 7:112–8. 10.23907/2017.013 31239963 PMC6474475

[18] Ying L Lin R Gao Z Qi J Zhang Z Gu W. Primary cardiac tumors in children: a center's experience. J Cardiothorac Surg. (2016) 11:52. 10.1186/s13019-016-0448-5 27067427 PMC4827228

[19] Pavlicek J Klaskova E Kapralova S Prochazka M Vrtel R Gruszka T. Fetal heart rhabdomyomatosis: a single-center experience. J Matern Fetal Neonatal Med. (2021) 34:701–7. 10.1080/14767058.2019.1613365 31032681

[20] Amelia A Mohd Nizam MB. Perinatal management of cardiac tumors: a case series. Med J Malaysia. (2013) 68:374–5. 24145275

[21] Roberts WC. Primary and secondary neoplasms of the heart. Am J Cardiol. (1997) 80:671–82. 10.1016/S0002-9149(97)00587-0 9295010

[22] Randhawa K Ganeshan A Hoey ET. Magnetic resonance imaging of cardiac tumors: part 2, malignant tumors and tumor-like conditions. Curr Probl Diagn Radiol. (2011) 40:169–79. 10.1067/j.cpradiol.2010.07.002 21616279

[23] Narin B Arman A Arslan D Simşek M Narin A. Assessment of cardiac masses: magnetic resonance imaging versus transthoracic echocardiography. Anadolu Kardiyol Derg. (2010) 10:69–74. 10.5152/akd.2010.016 20150010

[24] Ludomirsky A Vargo TA Murphy DJ Gresik MV Ott DA Mullins CE. Intracardiac undifferentiated sarcoma in infancy. J Am Coll Cardiol. (1985) 6:1362–4. 10.1016/S0735-1097(85)80226-6 4067117

[25] Mukai M Sameshima H Kodama Y Yamashita R Kaneko M Ikenoue T. Congenital infantile fibrosarcoma in a very low-birth-weight infant. J Pediatr Surg. (2012) 47:e1–4. 10.1016/j.jpedsurg.2011.11.052 22498408

